# Supplementary material for: High-Throughput Computational Screening of Two-Dimensional Covalent Organic Frameworks (2D COFs) for Capturing Radon in Moist Air
Source: Nanomaterials (Basel). 2023 May 3;13(9):1532. doi: 10.3390/nano13091532 (PMC10180264; doi:10.3390/nano13091532)
Supplement: Supplementary file 1 [file nanomaterials-13-01532-s001.zip › nanomaterials-2384205-supplementary.pdf]

nanomaterials

Supporting Information

High-Throughput Computational Screening of Two-Dimensional Covalent Organic Frameworks (2D COFs) for Capturing Radon in Moist Air

Hongyan Zeng [1], Xiaomin Geng [1], Shitong Zhang [2], Bo Zhou [3], Shengtang Liu [1,*] and Zaixing Yang [1,*]

[1] State Key Laboratory of Radiation Medicine and Protection, School for Radiological and Inter-Disciplinary Sciences (RAD-X), Collaborative Innovation Center of Radiation Medicine of Jiangsu Higher Education Institutions, Soochow University, Suzhou 215123, China; hyzeng@stu.suda.edu.cn (H.Z.); xmgengxmgeng@stu.suda.edu.cn (X.G.)

[2] State Key Laboratory of Separation Membranes and Membrane Processes, School of Chemical Engineering and Technology, Tiangong University, Tianjin 300387, China; zhangshitong@tiangong.edu.cn

[3] School of Big Data and Artificial Intelligence, Chengdu Technological University, Chengdu 611730, China; bozhou@zju.edu.cn

* Correspondence: liushengtang@suda.edu.cn (S.L.); zxyang@suda.edu.cn (Z.Y.)

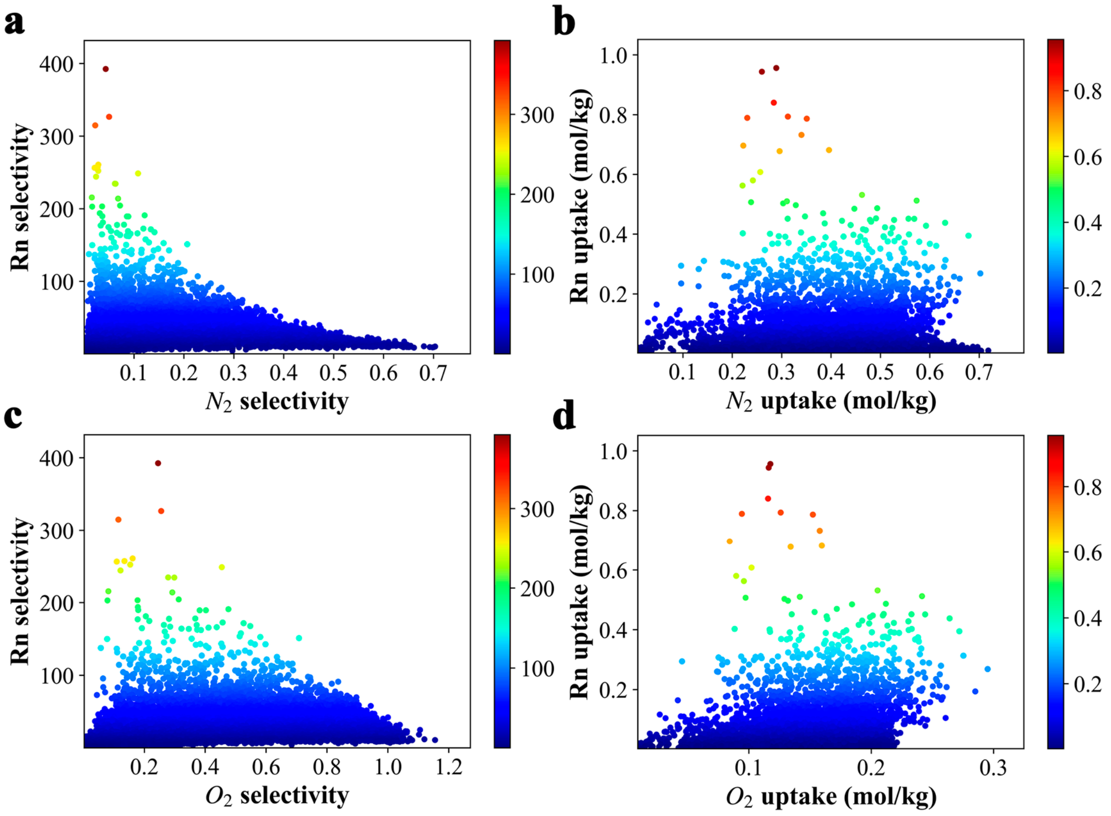

Figure S1. (**a**,**b**) Relationship between radon selectivity/uptake and nitrogen selectivity/uptake of COFs. (**c**,**d**) Relationship betwen radon selectivity/uptake and oxygen selectivity/uptake of COFs.

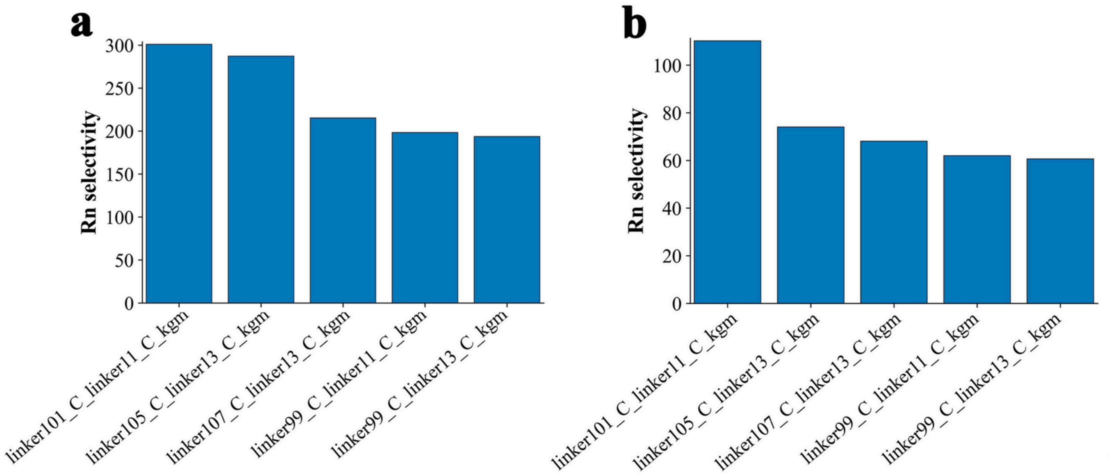

Figure S2. Rn selectivity order of the top five candidates obtained from two different force fields, **(a)** developed by Sanjon et al. [1] and **(b)** developed by Mick et al. [2].

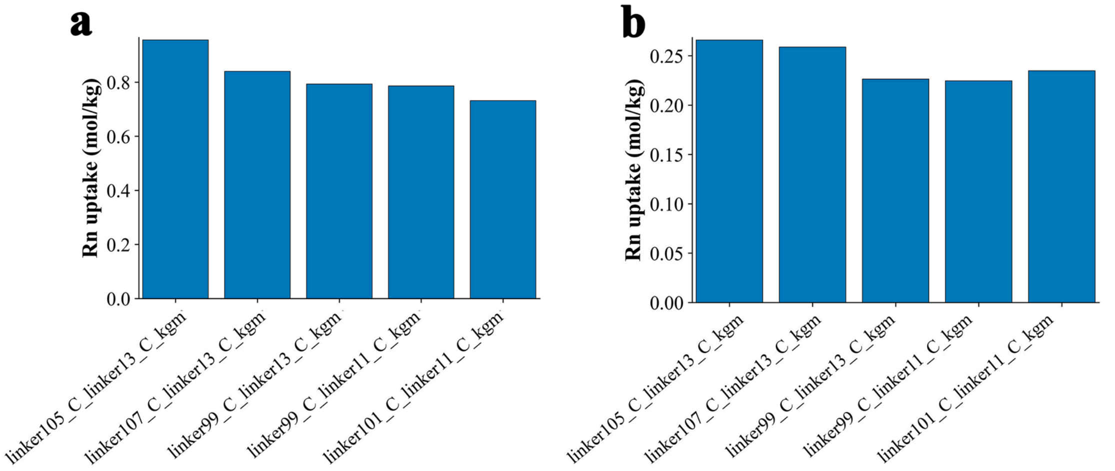

Figure S3. Rn uptake capacity order of the top five candidates obtained from two different force fields, **(a)** developed by Sanjon et al. [1] and **(b)** developed by Mick et al. [2].

Table S1. The IUPAC names and structures of the linker groups in top five COFs. Carbon atoms are shown in gray, nitrogen in light blue, hydrogen in white and Dummy atoms in green.

Linker #	IUPAC Name	Structure
11	3,6-dibromobenzene-1,2,4,5-tetraamine	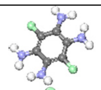
13	1,4-dibromo-2,3,5,6-tetramethylbenzene	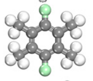
101	3,3’,5,5’-tetrabromo-2,2’-biphenyldiamine	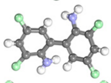
99	3,3,5,5-tetrabromo-1,1-biphenyl	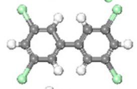
107	3,3’,5,5’-tetrabromo-4,4’-biphenyldiamine	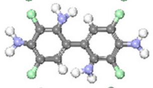
105	3,3’,5,5’-tetrabromo-4,4’-biphenyldiol	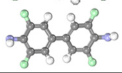

Table S2. Lennard-Jones Parameters of Rn atom in different force fields.

Model	σ (Å)	ε/k_b (K)
Sanjon et al. [1]	4.53	272.24
Mick et al. [2]	4.15	292.00

References

1. Sanjon, E.P.; Maier, A.; Hinrichs, A.; Kraft, G.; Drossel, B.; Fournier, C. A combined experimental and theoretical study of radon solubility in fat and water. *Sci. Rep.* **2019**, *9*, 10768. https://doi.org/10.1038/s41598-019-47236-y.
2. Mick, J.R.; Barhaghi, M.S.; Potoff, J.J. Prediction of radon-222 phase behavior by Monte Carlo simulation. *J. Chem. Eng. Data* **2016**, *61*, 1625–1631. https://doi.org/10.1021/acs.jced.5b01002.
